# Unpacking the impact of kindergarten organizational climate on teacher burnout: a latent profile analysis based on social systems theory and the JD-R model

**DOI:** 10.3389/fpubh.2025.1708777

**Published:** 2026-01-05

**Authors:** Liqun Wang, Honglei Li, Tianqi Qiao, Xinxin Wang, Pingzhi Ye, Jiaxin Xiang

**Affiliations:** 1 College of Education, Guangzhou University, Guangzhou, Guangdong, China; 2 Xuzhou Academy of Education Sciences, Xuzhou, Jiangsu, China

**Keywords:** organizational climate, burnout, latent profile analysis, kindergarten teachers, JD-R model

## Abstract

**Introduction:**

This study investigates the heterogeneity in kindergarten teachers’ perceptions of organizational climate and its impact on job burnout. Guided by the AGIL model from social systems theory and the Job Demands-Resources (JD-R) model, it addresses the need to move beyond variable-centered approaches to understand how distinct climate profiles are associated with teacher well-being.

**Methods:**

A person-centered latent profile analysis (LPA) was employed. A sample of 1,008 kindergarten teachers from China completed measures assessing organizational climate and burnout. The analysis aimed to identify distinct climate profiles and examine their relationships with demographic variables (kindergarten type, assessment level, teaching experience) and the three dimensions of burnout (emotional exhaustion, depersonalization, reduced personal accomplishment).

**Results:**

The LPA revealed five distinct organizational climate profiles: Controlled, Moderate, Indifferent, Positive, and Authoritative. Profile membership was significantly predicted by kindergarten assessment level and teachers’ years of experience, but not by kindergarten type. Crucially, the profiles differed significantly across all burnout dimensions. Teachers in Positive climates reported the lowest burnout levels, whereas those in Controlled and Indifferent climates experienced the highest.

**Discussion:**

The findings underscore the structural diversity of organizational climates in early childhood settings and their profound psychological consequences. This study validates the application of social systems theory and the JD-R model in this context, revealing how different configurations of job demands and resources shape teacher well-being. The results provide a theoretical lens for understanding educational organizations and offer practical implications for developing tailored, climate-specific intervention strategies to mitigate burnout and support sustainable professional development.

## 1 Introduction

### 1.1 Research background

In recent years, with the continuous advancement of early childhood education reform, preschool teachers have been facing increasing work-related challenges. Teacher burnout has emerged as a key issue hindering professional development and the improvement of educational quality. Burnout is a psychological syndrome resulting from prolonged exposure to chronic interpersonal stressors at work (1), and it is particularly prevalent among teachers (2). Compared to teachers at other educational levels, preschool teachers are more susceptible to higher levels of burnout due to factors such as the intensity of emotional labor, low social recognition, and frequent role conflicts (3). A substantial body of research has confirmed that burnout among preschool teachers not only undermines their teaching quality and job satisfaction but also significantly increases their intention to leave the profession, thereby potentially compromising children’s early learning environments and developmental opportunities (4, 5).

Furthermore, a comprehensive understanding of preschool teacher burnout must account for its heterogeneous and dynamic nature. Burnout is not a monolithic experience; it manifests differently across teachers based on their individual characteristics, such as career stage, professional resilience, and personal resources (6). The trajectory of burnout is often intertwined with teachers’ professional development cycles. Early-career teachers might experience burnout due to a lack of coping strategies and role ambiguity, while mid-career teachers might face burnout stemming from stagnation or excessive workload demands (7). This individual-differences perspective implies that the antecedents of burnout may also be perceived and experienced in meaningfully different ways by different teachers (8, 9).

Organizational climate, as an important contextual variable influencing teachers’ psychological states and behaviors, has received widespread attention in recent educational research. School organizational climate generally refers to teachers’ perceptions of their work environment (10). More specifically, climate is defined as a set of measurable attributes of the work environment that emerge from the collective perceptions of teachers and administrators (10). In the field of early childhood education, studies have shown that a positive organizational climate can alleviate teachers’ occupational stress and enhance their job satisfaction, professional engagement, and psychological well-being (11). In contrast, climates characterized by a lack of support, poor communication, or ambiguous rules and regulations have been empirically demonstrated to exacerbate emotional exhaustion and depersonalization among teachers, thereby intensifying their burnout levels (12).

Previous research has made significant efforts using variable-centered approaches to investigate the antecedents and outcomes of preschool organizational climate. For example, studies have found that organizational climate in preschools is positively associated with teacher job satisfaction and can positively predict instructional innovation (13, 14). These approaches help researchers understand the relationships among constructs of interest and formulate general intervention strategies. However, variable-centered approaches often overlook the existence of naturally occurring subgroups within the population (15) and only provide information on associations across the entire sample (16). Such limitations restrict deeper insight into the mechanisms through which organizational climate operates and hinder the development of tailored and targeted support strategies for teachers (15, 16).

To address these limitations, an increasing number of studies have adopted latent profile analysis (LPA) to identify perceptual heterogeneity within teacher groups. Unlike traditional variable-centered methods, LPA focuses on classifying teachers into several latent subgroups to reveal differences across key variables. This approach allows researchers to gain a more nuanced understanding of the characteristics of organizational climate subgroups in preschools and their associations with teacher burnout (17).

Therefore, the present study aims to employ LPA to systematically identify latent types of preschool teachers’ perceptions of organizational climate, and further examine the structural relationships between different perceptual profiles and burnout. The goal is to provide an empirical foundation for educational administrators to develop stratified support strategies, as well as to offer theoretical insights for optimizing the organizational environment in early childhood education and enhancing teacher wellbeing.

### 1.2 Theoretical framework

This study is underpinned by two foundational theories. The AGIL model, from Parsons’ social systems theory, offers a macro-sociological framework for analyzing how complex organizations maintain their structure and survive by addressing a set of fundamental, systemic prerequisites (18). The Job Demands-Resources (JD-R) model, in contrast, provides a parsimonious yet comprehensive psychological framework for understanding how the overall design and social-psychological aspects of any job can lead to either employee strain or motivation (19).

The AGIL model of social systems theory posits that a healthy organization can survive in its environment while continuously adapting to challenges and developing its capabilities (18). This survival and adaptability result from the necessary functions of social systems (20), specifically adaptation, goal attainment, integration, and pattern maintenance. Adaptation refers to acquiring resources and adapting to environmental changes; Goal Attainment involves setting and mobilizing resources to achieve goals; Integration emphasizes coordination and unity among system components; and Pattern Maintenance involves creating and maintaining unique value systems. These functions are key to maintaining the stability and adaptability of social systems (21).

This theory provides a new perspective for understanding kindergarten organizational climate. Specifically, kindergartens can be viewed as social systems with varying combinations of the four basic functions, forming different organizational climates. As highlighted in related research on organizational systems, disruptions in any of these functions can lead to systemic imbalances, manifesting in diminished organizational climate quality (22). A kindergarten with well-functioning adaptation, goal attainment, integration, and pattern maintenance is stable and responsive to challenges, exhibiting a positive organizational climate. Conversely, challenges in one or more functions can compromise stability and adaptability, negatively affecting the organizational climate. Previous research, such as that by Hoy et al. (20), has validated the applicability of this theoretical perspective in studying school organizational climates, underscoring its feasibility and relevance. Guided by this theory, this study explores the typical characteristics of different latent profiles of kindergarten organizational climate and seeks to uncover how various combinations of these functions shape diverse profiles.

To further explain the relationship between different perceived types of organizational climate and teacher burnout, this study introduces the JD-R model. The JD-R model posits that various work characteristics can be categorized into two broad dimensions: job demands and job resources. Job demands refer to aspects that require sustained effort and may lead to exhaustion, such as workload and emotional labor; in contrast, job resources are factors that help accomplish work goals, mitigate job demands, or foster personal development, such as organizational support, feedback, and autonomy.

The JD-R model outlines two core psychological processes. The health impairment process suggests that excessive job demands deplete individuals’ energy, resulting in fatigue and burnout. The motivational process indicates that job resources can stimulate positive motivation and enhance work engagement. The model also emphasizes that resources not only have direct motivational effects but also buffer the negative effects of high job demands, particularly under high-pressure conditions (23).

In early childhood education settings, organizational climate reflects teachers’ overall perceptions of support, control, and interpersonal relationships within the institution, essentially representing a configuration of job demands and resources. Different climate profiles (e.g., high support–low control or low support–high restriction) form distinct work environments that may influence levels of teacher burnout. The JD-R model thus provides a theoretical foundation for analyzing how organizational climate heterogeneity relates to teacher well-being.

### 1.3 Literature review

#### 1.3.1 The relationship between kindergarten organizational climate and teacher burnout

Empirical studies have shown that kindergarten teachers’ subjective perceptions of organizational climate play a critical role in their experience of burnout, with the two typically exhibiting a significant negative correlation (24). Specifically, a positive and harmonious organizational atmosphere can enhance teachers’ enthusiasm and motivation at work, enabling them to experience a greater sense of accomplishment and, consequently, lower levels of burnout. In contrast, when the school climate lacks trust and support, teachers are more likely to experience stress and disengagement, leading to heightened burnout levels (12). These findings highlight the importance of the organizational environment in alleviating teachers’ occupational stress and provide a solid empirical basis for the present study’s investigation of climate heterogeneity and its underlying mechanisms.

#### 1.3.2 Insights from person-centered approaches in organizational climate studies

Latent profile analysis, a person-centered statistical approach, has been increasingly applied in recent years to identify hidden patterns in teachers’ psychological and behavioral variables (25). At the organizational level, some studies have begun to apply LPA to analyze teachers’ perceptions of school climate. For example, Capp et al. (26) examined staff perceptions of school organizational climate across elementary, middle, and high schools in California and identified four latent profiles. Among them, staff in the positive climate profile reported the most favorable experiences; those in the positive discipline and support profile were more likely to evaluate the school’s disciplinary practices and support systems positively. In contrast, the negative climate profile was associated with the least favorable experiences, while the adverse climate profile reflected slightly better experiences than the negative one. These studies demonstrate that teachers’ subjective perceptions of the organizational environment may follow stable latent patterns, providing methodological support for identifying heterogeneity in climate perceptions.

However, due to limitations in sampling and measurement tools, the generalizability of such findings across different countries and regions remains uncertain. Moreover, organizational climate may vary across educational stages. Given that kindergartens differ significantly from primary and secondary schools in terms of environment, educational philosophy, and instructional approaches (27), their organizational climates and associated outcomes may also manifest in distinct ways. Therefore, it is necessary to apply a person-centered method to investigate the organizational climate in kindergarten settings.

#### 1.3.3 Demographic characteristics and kindergarten organizational climate

Previous studies on demographic characteristics and organizational climate largely focus on primary and secondary schools, with limited exploration of kindergartens. Li et al. (28) conducted a study involving 573 kindergarten teachers and discovered significant effects of kindergarten type (public or private) and assessment level (model kindergartens, first-class kindergartens, others) on organizational climate. Public kindergartens scored higher on principal support, limitations, and teacher engagement, while model kindergartens scored higher on principal support, teacher engagement, and intimacy. Public and model kindergartens tended to exhibit more positive organizational climates.

Expanding on these findings, studies have suggested that hierarchical assessment systems in education can exacerbate disparities in organizational climates. Clotfelter et al. (29) emphasized that rigid accountability measures often limit teacher autonomy and resource distribution, further influencing the organizational dynamics of educational institutions. These structural inequities call for more nuanced investigations to explore how such systems shape the organizational climates in early education. This aligns with findings from global studies emphasizing the role of institutional type and hierarchical assessment structures in shaping organizational climates. However, many studies in this domain have been constrained by sample homogeneity or a narrow focus on specific aspects of organizational climate, highlighting the need for a more comprehensive and nuanced approach.

Furthermore, existing research has paid limited attention to how early childhood teachers’ individual developmental characteristics (e.g., years of teaching experience, professional title) shape their perceptions of the organizational climate (30, 31). Crucially, from a person-centered perspective, these characteristics are not merely control variables; they are potential determinants of how teachers perceive and internalize their organizational environment (32). A novice teacher and a veteran expert, for instance, may interpret the same principal behavior as “supportive” or “controlling” in vastly different ways, leading them to psychologically inhabit different climate profiles even within the same kindergarten. Therefore, to fully unpack the heterogeneity of organizational climate and move beyond a one-size-fits-all understanding, it is essential to integrate these individual-level demographics into our analytical framework as core factors explaining profile membership.

### 1.4 The current study

**Figure 1:**
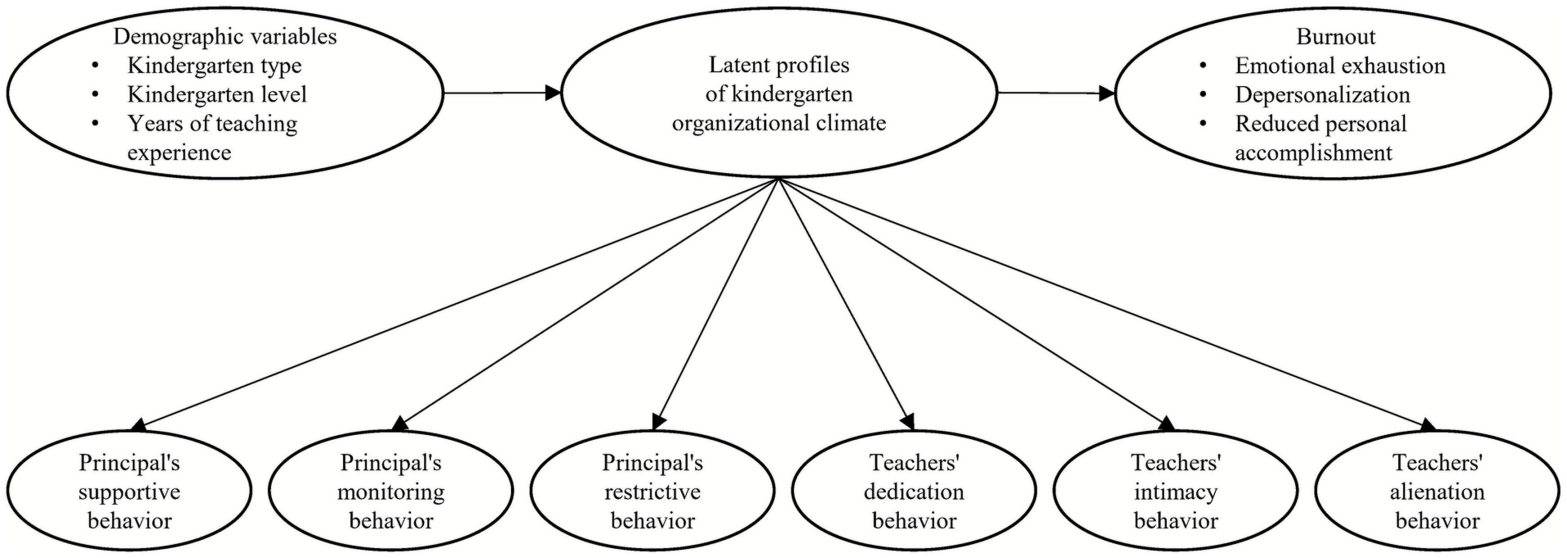
The hypothesized model.

Building on prior research on kindergarten organizational climate, the present study employed a person-centered approach to identify latent profiles of organizational climate in kindergartens. Second, we examined the associations between the identified organizational climate profiles and demographic variables, including kindergarten type, evaluation level and years of teaching experience. In addition, we investigated the relationships between different organizational climate profiles and dimensions of teacher burnout. Figure 1 presents the hypothesized model tested in this study.

RQ1: What distinct latent profiles of kindergarten organizational climate can be identified using a person-centered approach?

RQ2: How do these profiles differ in their associations with demographic variables?

RQ3: How do the identified organizational climate profiles differ in their associations with the various dimensions of teacher burnout?

By addressing these questions, this study aims to offer theoretical and practical insights that can enhance the quality and effectiveness of organizational climates in kindergartens.

## 2 Method

### 2.1 Participants

This study employed a convenience sampling method to collect data. We selected Guangdong and Jiangsu because both provinces are representative of different levels and models of early childhood education development, which enhances the external validity of the findings. In addition, existing collaborative partnerships in these regions ensured feasible and reliable data collection. In March 2025, online survey questionnaires were distributed to kindergarten teachers in Guangdong and Jiangsu provinces, China. The recruitment period lasted from March 10 to March 17, 2025. A total of 1,338 questionnaires were distributed, and 1,187 were returned. After excluding questionnaires with missing data, uniform responses for 85% of the items, or completion times under 5 min, 179 invalid questionnaires were removed. Ultimately, 1,008 valid responses were obtained, yielding a response rate of 84.9%. Table 1 presents the descriptive statistics of the participants’ demographic variables.

**Table 1:** Descriptive statistics of demographic variables of early childhood teachers.

Variable	Category	*N*	Percentage (%)/Mean (SD)	Range
Age	—	1,008	30.84 (6.61)	20.00–61.00
Gender	Male	8	0.8	—
	Female	1,000	99.2	—
Kindergarten type	Public	837	83.0	—
	Private	171	17.0	—
Kindergarten assessment level	Province and city level	564	56.0	—
	County and district level	304	30.2	—
	Unrated	140	13.9	—
Years of teaching experience	0–5 years	331	32.8	—
	6–10 years	412	40.9	—
	>10 years	265	26.3	—

N, number; SD, standard deviation.

The study complied with the ethical principles of voluntary participation and the Declaration of Helsinki, obtaining informed consent. Before completing the questionnaire, kindergarten teachers were informed that their data would be anonymized and confidential, and they were asked if they wished to participate. If they agreed, the questionnaire was presented; otherwise, the survey was terminated. Participants could withdraw at any time. The study was approved by the ethics committee of the first author’s institution.

### 2.2 Measures

#### 2.2.1 Personal information questionnaire

The personal information questionnaire collected data on teachers’ gender, age, years of teaching experience (0–5, 6–10, and >10 years), kindergarten type (public or private), and kindergarten assessment level (province and city level, county and district level, or unrated).

#### 2.2.2 Organizational climate

The Chinese-adapted version of the Kindergarten Organizational Climate Scale was used (28). Initially developed by Croft and Halpin (33) in 1962, the scale has been widely used to measure organizational climate in schools. Hoy and Clover (10) revised the OCDQ questionnaire to address its limitations, and it has since been applied across various countries and cultures, demonstrating good psychometric properties (34, 35). The revised scale, known as the OCDQ-RE, includes six dimensions encompassing principal and teacher behaviors, with 33 items (e.g., “The principal listens to and accepts teachers’ suggestions”; “The principal monitors everything teachers do.”). Each item is rated on a 5-point Likert scale (1 = strongly disagree, 5 = strongly agree). All items except two negatively scored items (e.g., “Teachers rarely help or support each other”) are positively scored. The scale has shown good reliability and validity in Chinese populations (36). In this study, the scale’s Cronbach’s alpha coefficient was 0.75.

#### 2.2.3 Teacher burnout

Teacher burnout was measured using the Chinese-adapted version of the Maslach Burnout Inventory–Educators Survey (MBI-ES) (37). The original scale was developed by Maslach and Jackson (38) in 1981 and consists of 22 items across three dimensions: emotional exhaustion, depersonalization, and reduced personal accomplishment. Example items include “I feel emotionally drained from my work” and “I feel used up at the end of the workday.” All items are rated on a 5-point Likert scale (1 = strongly disagree, 5 = strongly agree), with all items scored in the positive direction. The scale has demonstrated good reliability and validity in Chinese populations (39). In the present study, the Cronbach’s *α* coefficient for the scale was 0.96.

### 2.3 Data analysis

Data were collected through online questionnaires, with no missing values identified, thus eliminating the need for missing data imputation. SPSS 27.0 was used for data management, descriptive statistics, multinomial logistic regression analysis and analysis of variance (ANOVA).

To address RQ1, LPA was conducted using the tidyLPA package in R (40), a categorical latent variable modeling method, identifies subgroups within a population based on a set of variables (25, 41). The tidyLPA package facilitates LPA execution, allowing specification of different models and the number of profiles to be estimated. Table 2 presents detailed descriptions of each model. Model fit indices, including Akaike’s information criterion (AIC), Bayesian information criterion (BIC), Sample size-adjusted Bayesian information criterion (SABIC), entropy, and the Bootstrap likelihood ratio test (BLRT), were assessed to identify the best-fitting model. Lower AIC, BIC, and SABIC values indicate better model fit; entropy values closer to 1 indicate more accurate classification, while significant BLRT results (*p* < 0.05) suggest that the n-profile solution fits better than the n-1 profile solution. Solutions with rare categories (<5% of the sample) were excluded due to challenges in replicability (42, 43). The interpretability of profile solutions was also considered.

**Table 2:** TidyLPA model and its characteristics (Rosenberg (53)).

Model	Alternative name	Variances	Covariances	Characteristics
1	Class-invariant parameterization	Equal	Fixed to 0	Highly constrained but also parsimonious.
2	Class-varying diagonal parameterization	Varying	Fixed to 0	More flexible (and less parsimonious) than model 1.
3	Class-invariant unrestricted parameterization	Equal	Equal	Adding more information that can be used to better understand the characteristics of the profiles (and, potentially, better explain the data).
6	Class-varying unrestricted parameterization	Varying	Varying	Although it is the most complex model, it is less parsimonious than all the other models.

To address RQ2, multinomial logistic regression was employed to examine the effects of kindergarten type, assessment level and years of teaching experience on organizational climate profiles. To address RQ3, ANOVA was conducted to assess the associations between each kindergarten organizational climate profile and teacher burnout.

## 3 Results

### 3.1 LPA results: profile composition

To address Research Question 1, LPA was conducted to explore the organizational climate in kindergartens. The objective was to identify the model that best represents the data by comparing and evaluating the fit indices of various models (see Table 3). Models 2 and 6 were excluded as their data failed to converge. Consequently, Models 1 and 3 were compared. Among the AIC, BIC, and SABIC indices, Model 3 outperformed Model 1. However, the four-profile solution in Model 3 had a low entropy value (0.71), and the smallest class in the five-profile solution contained less than 5% of the sample, rendering it impractical. While the three-profile solution offered favorable fit indices, its classification lacked interpretability. Furthermore, Model 3’s unconstrained covariance could lead to parameter estimation instability, whereas Model 1 employed a fixed covariance of zero, providing greater structural simplicity. Considering these factors, Model 1 was selected for further analysis. The five-profile solution within Model 1 demonstrated the lowest AIC, BIC, and SABIC values, the highest entropy value (0.94), and a significant BLRT, indicating its superiority over the four-profile solution. Therefore, the five-profile solution of Model 1 was chosen for subsequent analysis.

**Table 3:** Model fit indices for the latent profile analysis.

Model	Classes	AIC	BIC	SABIC	Entropy	N_Min	BLRT_p
1	1	94498.13	94822.56	94612.94	1.00	1.00	
1	2	89135.77	89627.34	89309.74	0.87	0.39	<0.01
1	3	84711.71	85370.41	84944.82	0.97	0.17	<0.01
1	4	83432.26	84258.10	83724.52	0.93	0.14	<0.01
**1**	**5**	**82641.31**	**83634.28**	**82992.72**	**0.94**	**0.06**	**<0.01**
3	1	78797.50	81717.44	79830.86	1.00	1.00	
3	2	78390.89	81477.97	79483.40	0.97	0.14	<0.01
3	3	77060.71	80314.92	78212.36	1.00	0.08	<0.01
3	4	76879.26	80300.60	78090.05	0.71	0.11	<0.01
3	5	76772.50	80360.98	78042.45	0.81	0.04	<0.01

Model fit statistics from 5 LPA models (1–5 profiles). AIC, akaike information criteria; BIC, Bayesian information criteria; SABIC, sample size-adjusted Bayesian information criteria; BLRT_p, *p*-value for the bootstrapped likelihood ratio test. Boldened row indicates the selected solution.

**Figure 2:**
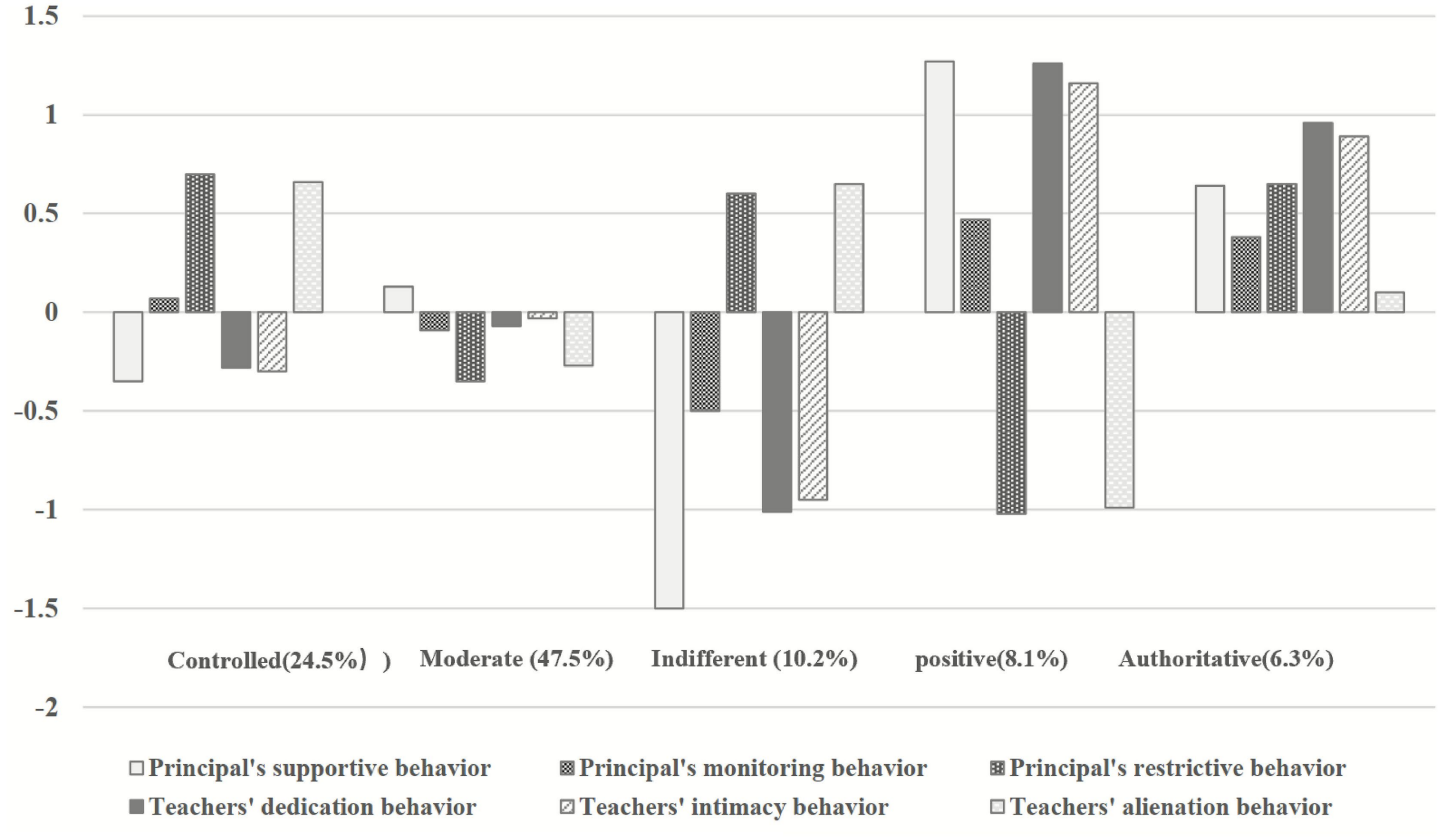
Standardized mean estimates of kindergarten organizational climate.

Table 4 presents the means, standard deviations, and *post hoc* comparisons of the profiles, while Figure 2 illustrates the standardized estimates of the analysis (*z*-scores). In terms of profile interpretation, Profile 1 consisted of 247 participants, accounting for 24.5% of the total sample. Compared to other profiles, kindergarten teachers in Profile 1 perceived greater restrictions from principals, and this profile was labeled as the “Controlled Organizational Climate.” The majority of participants (*n* = 479), comprising 47.5% of the total sample, were categorized into Profile 2. This profile exhibited average levels across all dimensions and was thus labeled as the “Moderate Organizational Climate.” Profile 3 included 103 teachers, accounting for 10.2% of the total sample. Teachers in this profile perceived little support and supervision from principals but experienced more restrictions, resulting in low engagement and intimacy. This profile was termed the “Indifferent Organizational Climate.” Additionally, Profile 4 consisted of 115 teachers, representing 11.4% of the total sample. This profile featured high support, moderate supervision, and minimal restrictions from principals, as well as high teacher engagement and intimacy alongside low levels of alienation. Hence, this profile was labeled as the “Positive Organizational Climate.” Finally, Profile 5 included 64 teachers, accounting for 6.3% of the total sample. This profile reflected a combination of high support and high restrictions from principals, with high levels of teacher engagement and intimacy. This profile was labeled as the “Authoritative Organizational Climate” (see Table 5).

**Table 4:** Latent profile analysis of organizational climate.

	1. Controlled (*n* = 247)	2. Moderate (*n* = 479)	3. Indifferent (*n* = 103)	4. Positive (*n* = 115)	5. Authoritative (*n* = 64)	
Variables			M (SD)			Comparison
Principal’s supportive behavior	3.54 (0.39)	3.93 (0.24)	2.68 (0.61)	4.80 (0.30)	4.30 (0.55)	4>5>2>1>3
Principal’s monitoring behavior	3.67 (0.38)	3.51 (0.40)	3.23 (0.50)	3.91 (0.67)	3.88 (0.58)	4>1>2>3, 5>2>3
Principal’s restrictive behavior	3.64 (0.46)	2.58 (0.52)	3.52 (0.65)	1.91 (0.65)	3.59 (0.70)	1 = 3 = 5>2>4
Teachers’ dedication behavior	3.88 (0.21)	4.01 (0.16)	3.44 (0.37)	4.80 (0.22)	4.62 (0.22)	4>5>2>1>3
Teachers’ intimacy behavior	3.69 (0.33)	3.91 (0.27)	3.24 (0.43)	4.73 (0.33)	4.51 (0.41)	4>5>2>1>3
Teachers’ alienation behavior	3.10 (0.48)	2.23 (0.34)	3.06 (0.50)	1.59 (0.50)	2.59 (0.73)	1 = 3>5>2>4

“>” denotes that the latter cluster has a significantly lower score than the former cluster at *p* < 0.05.

**Table 5:** Summary of key characteristics of the five identified organizational climate profiles.

Profile name (Label)	Sample size (*n*, %)	Key characteristics across dimensions
1. Controlled	247 (24.5%)	- High principal restrictive behavior - Moderate principal supportive/monitoring behavior - Moderate teacher dedication and intimacy - High teacher alienation
2. Moderate	479 (47.5%)	- Moderate levels across all six dimensions - A balanced, average perception of the climate.
3. Indifferent	103 (10.2%)	- Low principal supportive and monitoring behavior - High principal restrictive behavior - Low teacher dedication and intimacy - High teacher alienation
4. Positive	115 (11.4%)	- High principal supportive behavior - Moderate principal monitoring behavior - Low principal restrictive behavior - High teacher dedication and intimacy - Low teacher alienation
5. Authoritative	64 (6.3%)	- High principal supportive behavior - High principal restrictive behavior - High teacher dedication and intimacy - Moderate teacher alienation

The characteristics are based on the mean scores and comparisons reported in Table 4 and the narrative description in Section 3.1.

### 3.2 Relationships between demographic variables and kindergarten organizational climate profiles

To address Research Question 2, the relationships between demographic variables and the five identified latent profiles were analyzed (see Table 6). The results indicated that kindergarten type was not significantly associated with the latent profiles. However, kindergarten assessment level demonstrated significant associations with the profiles. Specifically, teachers working in provincial and municipal kindergartens were more likely to perceive positive (*p* < 0.01), moderate (*p* < 0.05), and authoritative (*p* < 0.05) organizational climates compared to those in unrated kindergartens, which were more associated with controlled climates. The results further showed that teaching experience significantly predicted teachers’ organizational climate profiles. This effect was concentrated in the comparison between teachers with 0–5 years of experience and those with more than 10 years, whereas no significant differences emerged between the 6–10-year group and the >10-year group across any profile contrasts. Relative to teachers with over 10 years of experience, those with 0–5 years of experience demonstrated a markedly lower probability of being characterized by an authoritative rather than a positive climate (*p* < 0.05) and were also less likely to be associated with an authoritative instead of a moderate climate (*p* < 0.05). In addition, teachers in the early career stage showed reduced odds of being situated in a Controlled rather than an indifferent climate (*p* < 0.05), and the likelihood of being embedded in an authoritative context instead of an indifferent one was even further diminished (*p* < 0.01).

**Table 6:** Predictors of organizational climate profiles.

Variables	b (SE)	OR	b (SE)	OR	b (SE)	OR	b (SE)	OR	b (SE)	OR
	Controlled vs. positive (Re)		Moderate vs. positive (Re)		Indifferent vs. positive (Re)		Authoritative vs. positive (Re)		Controlled vs. moderate (Re)	
Kindergarten type^a^	−0.14 (0.35)	0.87	−0.56 (0.32)	0.57	−0.65 (0.39)	0.52	−0.26 (0.47)	0.77	0.42 (0.22)	1.52
Kindergarten assessment level										
Province and city level	−1.15** (0.40)	**0.32**	−0.62 (0.38)	0.54	−0.64 (0.47)	0.53	0.01 (0.60)	1.01	−0.53* (0.23)	**0.59**
County and district level	−0.59 (0.43)	0.56	−0.27 (0.42)	0.77	−0.13 (0.51)	0.88	0.17 (0.64)	1.18	−0.32 (0.24)	0.72
Unrated	—	—	—	—	—	—	—	—	—	—
Years of teaching experience										
0–5 years	−0.19 (0.31)	0.83	0.02 (0.28)	1.02	0.46 (0.37)	1.58	−0.95* (0.46)	0.39	−0.21 (0.21)	0.82
6–10 years	−0.23 (0.28)	0.79	−0.34 (0.26)	0.71	−0.06 (0.36)	0.95	−0.14 (0.37)	0.87	0.11 (0.19)	1.12
>10 years	—	—	—	—	—	—	—	—	—	—

Variables	Indifferent vs. Moderate (Re)		Authoritative vs. Moderate (Re)		Controlled vs. Indifferent (Re)		Authoritative vs. Indifferent (Re)		Controlled vs. Authoritative (Re)	
Kindergarten type^a^	−0.09 (0.28)	0.92	0.29 (0.38)	1.34	0.51 (0.31)	1.66	0.38 (0.44)	1.47	0.13 (0.41)	1.14
Kindergarten assessment level										
Province and city level	−0.02 (0.34)	0.98	0.63 (0.50)	1.88	−0.51 (0.35)	0.60	0.65 (0.57)	1.92	−1.16* (0.51)	**0.31**
County and district level	0.14 (0.36)	1.15	0.43 (0.53)	1.54	−0.46 (0.38)	0.63	0.30 (0.61)	1.34	−0.76 (0.55)	0.47
Unrated	—	—	—	—	—	—	—	—	—	—
Years of teaching experience										
0–5 years	0.44 (0.29)	1.55	−0.97* (0.41)	0.38	−0.65* (0.32)	0.52	−1.41** (0.47)	0.24	0.76(0.42)	2.15
6–10 years	0.29 (0.29)	1.33	0.20 (0.30)	1.22	−0.18 (0.31)	0.84	−0.09 (0.39)	0.92	−0.09(0.32)	0.92
>10 years	—	—	—	—	—	—	—	—	—	—

Re, Reference Group; OR, odds ratio. Significant results are indicated in bold. *p < 0.05, **p < 0.01, ***p < 0 0.001.

a Kindergarten type was coded 1 for public and 2 for private.

### 3.3 The relationship between kindergarten organizational climate profiles and teacher burnout

To address Research Question 3, we examined the associations between the identified kindergarten organizational climate profiles and teacher burnout. Table 7 presents the means, standard errors, and *post hoc* comparison results. Significant chi-square statistics were observed for each outcome: emotional exhaustion [χ^2^(4) = 170.661, *p* < 0.001], depersonalization [χ^2^(4) = 169.515, *p* < 0.001], and reduced personal accomplishment [χ^2^(4) = 175.202, *p* < 0.001]. Further pairwise comparisons across climate profiles revealed that teachers in the controlled and indifferent climate profiles reported significantly higher levels of emotional exhaustion, depersonalization, and reduced personal accomplishment than those in the moderate, authoritative, and positive profiles. Additionally, teachers in the moderate and authoritative profiles reported significantly higher levels of burnout across all three dimensions compared to those in the positive profile. No significant differences were found between the controlled and indifferent profiles, nor between the moderate and authoritative profiles, in terms of their effects on the three dimensions of teacher burnout.

**Table 7:** Distal outcomes of latent profiles of organizational climate.

	Controlled (*n* = 247)	Moderate (*n* = 479)	Indifferent (*n* = 103)	Positive (*n* = 115)	Authoritative (*n* = 64)	
Variables			M (S. E.)			Comparison
Emotional Exhaustion	2.74 (0.04)	2.16 (0.02)	2.90 (0.06)	1.50 (0.04)	2.13 (0.08)	1 = 3>2 = 5>4
Depersonalization	2.58 (0.04)	2.03 (0.02)	2.75 (0.07)	1.35 (0.05)	1.86 (0.09)	1 = 3>2 = 5>4
Reduced Personal Accomplishment	2.58 (0.05)	2.04 (0.02)	2.83 (0.07)	1.35 (0.05)	1.98 (0.09)	1 = 3>2 = 5>4

Overall chi-squared statistics were statistically significant (*p* < 0.001). “>” denotes that the latter cluster has a significantly lower score than the former cluster at *p* < 0.05.

## 4 Discussion

This study focused on the latent heterogeneity of kindergarten organizational climate and identified several psychologically meaningful profile types. It further examined the associations between these profiles and institutional characteristics, including kindergarten type and evaluation level. Building on this, the study systematically investigated how different climate profiles relate to the various dimensions of teacher burnout. By integrating latent profile analysis with burnout indicators, this research is among the first to reveal the structural differences in organizational climate and their impact on emotional exhaustion, depersonalization, and reduced personal accomplishment. The findings offer a valuable extension to previous variable-centered research on organizational climate (2, 44, 45). Moreover, they deepen our understanding of the complex structure and psychological consequences of kindergarten organizational climate, providing empirical support for the development of targeted strategies to optimize school climate and support teacher wellbeing.

### 4.1 Latent profiles of kindergarten organizational climate

This study identified five distinct latent profiles of kindergarten organizational climate. The findings align partially with prior research, suggesting that kindergarten organizational climate comprises both positive and negative aspects (27, 30). The results resonate with the four basic functions of social systems theory—adaptation, goal-attainment, integration, and pattern-maintenance—and their role in maintaining the stability and adaptability of social systems. Specifically, the positive organizational climate represents the most ideal state, characterized by close collaboration and alignment among various organizational components, leading to a highly unified value system. Teachers in this climate maintain high levels of enthusiasm, efficiency, and a strong sense of recognition and belonging within the organization.

Conversely, the indifferent organizational climate exhibited the most adverse outcomes among the five profiles. Although emphasizing goal achievement, this climate lacks necessary resources and support, leading to poor coordination among organizational components. Teachers in this environment often experience significant work stress, fatigue, and helplessness. The moderate organizational climate remains stable across the four functions but falls short of achieving the standards of a highly efficient social system. Teachers in this climate perceive a moderate level of intervention and autonomy from directors, maintaining a balanced enthusiasm for work and professional development while establishing appropriate boundaries in colleague relationships.

Additionally, the study identified controlled and authoritative organizational climates. In the controlled organizational climate, the kindergarten system ensures functional operations but often emphasizes goal attainment excessively, imposing numerous restrictions on teachers, such as assigning extensive documentation and reporting tasks. This climate results in highly controlled activities and increased stress and resistance among teachers. Similarly, an authoritative organizational climate often imposes numerous restrictions during the process of setting and achieving goals. However, this type of climate also provides ample resource support alongside these restrictions, thereby enabling the organization to maintain good coordination internally. Teachers face significant pressure and challenges but can leverage resources to enhance their professional capabilities and achieve organizational goals. The prevalence of these control-oriented climates may reflect the sociocultural context of China, where education systems are characterized by centralization and hierarchical management (46). Under these circumstances, strict management emphasizing standardization and quality control is prevalent. However, overly detailed and stringent management practices may induce psychological pressure on teachers, such as through last-minute environmental adjustments and preparations for administrative inspections, which can disrupt regular educational activities. These findings highlight the need to balance control with support to create healthier organizational climates.

### 4.2 Relationships between demographic variables and organizational climate profiles

First, the findings revealed no significant relationship between kindergarten type and organizational climate profiles, contradicting previous research (28). This discrepancy may stem from the multifaceted nature of organizational climate, influenced by various factors such as leadership styles and teacher attitudes (10). These factors are present in both public and private kindergartens, exhibiting significant variability and plasticity across different institutions. Furthermore, in the context of advancing high-quality early childhood education, both public and private kindergartens are required to comply with relevant standards and regulations, limiting substantial differences in organizational climate.

Second, the study found that teachers in provincial or municipal kindergartens were more likely to experience positive, authoritative, and moderate organizational climates than controlled climates, compared to those in unrated kindergartens. This aligns with previous research and can be attributed to differences in support and resources (28). Specifically, provincial or municipal kindergartens often have better material resources and professional foundations, attracting quality students and receiving more educational resources and financial support. In contrast, unrated kindergartens may face challenges such as limited resources and inconsistent management practices, leading administrators to adopt controlling measures to seek educational outcomes, thereby fostering a restrictive climate. These findings further validate the heterogeneity of kindergarten organizational climate and provide evidence for investigating the impact of demographic variables.

Relative to teachers with more than 10 years of experience, those with 0–5 years show lower odds of belonging to the authoritative profile (vs. Positive, Moderate, or Indifferent) and to the Controlled profile (vs. Indifferent). A likely explanation is that early-career teachers have not yet developed full role clarity, familiarity with organizational routines, or reliable access to support resources, so feedback and monitoring are more easily perceived as external pressure rather than developmental support (23, 47). Their instructional and management efficacy is also still consolidating, which makes job demands more salient than available resources, consistent with JD–R theory and career stage models (7, 23).

The lack of significant contrasts between 6 and 10 years and >10 years suggests convergence after teachers learn the school’s routines and expectations: both groups achieve similar balances between what is asked of them and what they can draw on (time, materials, collegial advice, principal support), and they interpret leadership signals in more similar ways, yielding stable and comparable profile membership.

### 4.3 The impact of kindergarten organizational climate on teacher burnout

This study examined the impact of different latent profiles of kindergarten organizational climate on teacher burnout and found that climate heterogeneity significantly predicted emotional exhaustion, depersonalization, and reduced personal accomplishment. This finding not only supports the core assumption of the dual-pathway mechanism proposed by the JD-R model but also offers a new perspective for understanding how differences in resource allocation within educational settings shape psychological outcomes.

Results showed that teachers in the positive climate profile reported the lowest levels of burnout, consistent with previous findings (10, 12). In this climate type, principals provided high levels of emotional support, moderate supervision, and minimal control, while teachers demonstrated strong engagement and close interpersonal relationships. This “high support–high interaction–low control” configuration created a resource-rich and structurally balanced work environment that fulfilled teachers’ basic psychological needs for autonomy, relatedness, and competence. It also enhanced their work engagement and psychological resilience, thereby effectively mitigating burnout (48).

In contrast, teachers in the controlled and indifferent climate profiles exhibited significantly higher levels of burnout. The indifferent profile was characterized by a systemic lack of resources—principals offered minimal support or supervision while exercising high levels of control, and teacher engagement and collegial intimacy were extremely low. This absence of key supportive resources reflects a typical health impairment process as outlined in the JD-R model (23). Although the controlled climate showed slightly more favorable levels of support and teacher behaviors compared to the indifferent type, its strong emphasis on restrictive management may have psychologically undermined the limited resources available, rendering them ineffective in buffering high job demands (49, 50). Notably, no significant differences in burnout levels were found between the Controlled and Indifferent profiles. This finding can be elucidated through the health impairment process of the JD-R model. Both profiles are ultimately defined by a critical insufficiency of functional job resources. In the Indifferent climate, the absence of principal support, supervision, and collegial intimacy creates a fundamentally resource-deprived environment. In the Controlled climate, although marginally higher levels of support and teacher engagement are present, the pervasive and psychologically taxing job demands—such as restrictive management and intensive monitoring—are likely to override and neutralize the potential buffering effects of these limited resources (23). Consequently, teachers in both climate types arguably operate in a state of chronic resource depletion, leading to similarly elevated levels of emotional exhaustion, depersonalization, and reduced personal accomplishment.

Furthermore, the lack of a significant burnout difference between the Moderate and Authoritative profiles underscores a more complex, counterbalancing dynamic. The Authoritative profile is indeed resource-rich, characterized by high principal support and strong teacher collegiality. However, these resources are coupled with comparably high levels of restrictive control, which constitutes a significant job demand. We posit that the motivational benefits afforded by the abundant resources are partially offset or “neutralized” by the psychological costs associated with the demands for compliance and control, a phenomenon consistent with the complex interplay within the JD-R model (51). The Moderate profile, in contrast, presents a more balanced but unexceptional configuration of moderate demands and resources. The net psychological effect—the balance between the costs of demands and the benefits of resources—may thus be comparable between these two distinct profiles, resulting in non-significant differences in overall burnout levels. Together, these findings highlight the non-linear and interactive nature of how different organizational climate characteristics combine to shape teacher wellbeing.

### 4.4 Theoretical and practical implications

#### 4.4.1 Theoretical implications

This study offers meaningful theoretical contributions to the field of early childhood education and organizational research. First, by drawing on the AGIL framework from social systems theory, the study provides a novel and systematic lens for interpreting the latent profiles and structural characteristics of kindergarten organizational climate. This approach not only extends the applicability of the AGIL model to the kindergarten context but also enriches our understanding of how kindergartens, as subsystems of society, strive to maintain functional balance and adapt to evolving educational environments. It addresses a long-standing gap in organizational climate research in education, which has often lacked a clear theoretical foundation.

Second, by employing a person-centered approach, the study identifies distinct subgroups in teachers’ perceptions of organizational climate, thus revealing the heterogeneity embedded within staff experiences. This challenges the traditional view of organizational climate as a homogeneous construct and offers a theoretical basis for developing more differentiated and targeted organizational management strategies. Furthermore, the study explores how demographic variables, such as kindergarten type and evaluation level, influence climate profile membership. These findings both validate and nuance prior research and lay the groundwork for future investigations into the demographic determinants of perceived organizational climate.

#### 4.4.2 Practical implications

The findings of this study also have important practical relevance. First, identifying distinct profiles of kindergarten organizational climate enables school leaders and administrators to better recognize the varying needs and expectations of teaching staff, thereby informing the design of more personalized professional development and motivational strategies. Second, in terms of demographic variables, the results indicate that teachers in provincial- and municipal-level kindergartens are more likely to perceive their organizational climate as positive, authoritative, or moderate, rather than as control-oriented. Based on these findings, high-rated kindergartens can adopt climate-specific strategies to consolidate existing strengths, while unrated kindergartens should prioritize addressing the core issues associated with control-type climates. This may include implementing leadership training, enhancing teacher empowerment, and promoting more democratic and participatory leadership styles in order to mitigate excessive top-down control and foster a more supportive and resilient organizational environment. In light of the observed links between teaching experience and organizational-climate profiles, kindergartens should implement a tenure-tiered support system. Specifically, for teachers with 0–5 years of experience, provide structured mentoring, standardized onboarding to school routines, and clearly identified help channels so that norms and monitoring are framed as developmental support; for teachers with 6–10 and >10 years, prioritize collaborative empowerment and process streamlining to prevent excessive demands and to sustain stable membership in higher-quality climate profiles.

### 4.5 Limitations and future research directions

This study has several limitations that warrant consideration. First, the reliance on self-reported data from a single source (teachers) at a single time point introduces the potential for common method variance, which could inflate the relationships between variables (52). While we ensured anonymity to reduce social desirability bias, this limitation should be considered when interpreting the results. Future research could enhance reliability and validity by employing mixed-method approaches, including observational data, interviews, and third-party assessments, to triangulate findings. Second, the study utilized a convenience sampling method, drawing participants from only two provinces, Guangdong and Jiangsu. This limitation calls for caution in generalizing the findings to broader populations. Future studies should aim to include samples from diverse geographical regions and varied educational contexts to improve the representativeness and applicability of results. Lastly, the cross-sectional design of this study limits its ability to examine dynamic changes in organizational climate over time. To address this, longitudinal research designs employing growth models or cross-lagged panel models are recommended for future studies to explore causal relationships and temporal dynamics among variables, providing a more comprehensive understanding of kindergarten organizational climate.

## 5 Conclusion

Guided by social systems theory and the JD-R model, this study revealed five distinct profiles of kindergarten organizational climate and demonstrated their differential impacts on teacher burnout. Positive climates were linked to the lowest burnout levels, while controlled and indifferent climates were associated with the highest. The findings underscore the importance of balancing support and control to foster teacher well-being. These results offer a theoretical framework and empirical basis for differentiated interventions and climate optimization in early childhood education settings.

## Data Availability

The datasets generated and/or analyzed during the current study are available from the corresponding author on reasonable request.
