# Correction: Unpacking the impact of kindergarten organizational climate on teacher burnout: a latent profile analysis based on social systems theory and the JD-R model

**DOI:** 10.3389/fpubh.2026.1794384

**Published:** 2026-02-16

**Authors:** Liqun Wang, Honglei Li, Tianqi Qiao, Xinxin Wang, Pingzhi Ye, Jiaxin Xiang

**Affiliations:** 1 College of Education, Guangzhou University, Guangzhou, Guangdong, China; 2 Xuzhou Academy of Education Sciences, Xuzhou, Jiangsu, China

**Keywords:** organizational climate, burnout, latent profile analysis, kindergarten teachers, JD-R model

An incorrect Funding statement was provided. The correct funding statement reads:

The author(s) declared that financial support was received for this work and/or its publication. This study was supported by the Philosophy and Social Science Planning Project of Guangdong Province (Grant No. GD24CJY08).

The original version of this article has been updated.

